# The effect of health literacy, self-efficacy, social support and fear of disease progression on the health-related quality of life of patients with cancer in China: a structural equation model

**DOI:** 10.1186/s12955-023-02159-1

**Published:** 2023-07-18

**Authors:** Ling Zhang, Yumei Shi, Jing Deng, Dali Yi, Ji-an Chen

**Affiliations:** 1 Department of Health Education, College of Military Preventive Medicine, Army Medical University, Chongqing, 400038 China; 2 Chongqing University Cancer Hospital, Chongqing, 400030 China; 3 Gastric Cancer Center, West China Hospital, Sichuan University, Sichuang, 610041 China

**Keywords:** Health literacy, Health-related quality of life, Structural equation model, Self-efficacy, Social support, Fear of disease progression

## Abstract

**Background:**

Health literacy (HL), self-efficacy (SE), social support (SS) and fear of disease progression (FOP) are all important factors affecting health-related quality of life (HRQoL) in cancer patients. However, their synergistic effects and underlying mechanisms on HRQoL in cancer patients remain unclear. Therefore, the purpose of this study was to construct a structural equation model (SEM) to explore the underlying mechanism of factors affecting HRQoL. It is hoped that this study will provide a theoretical basis for future interventions.

**Methods:**

A cross-sectional design and convenience sampling method were used to investigate cancer inpatients in two general hospitals in Chongqing and Chengdu. Data were collected using structured scales, including HL, SE, SS, FOP and HRQoL. Finally, the SEM was constructed, and *P* ≤ 0.05 was considered significant.

**Results:**

There were 1749 participants included in this study. Correlation analysis showed that all variables were significantly correlated with one another except for symptoms, physical health (PD) and social family (SF) (*p* < 0.01). The SEM of the HRQoL had a good overall fit (GFI = 0.943, AGFI = 0.917, NFI = 0.950, RFI = 0.936, CFI = 0.955, IFI = 0.955, RMSEA = 0.072). The model indicated that HL had the strongest correlation with HRQoL (β = 0.398, *p* < 0.01), followed by FOP (β = -0.364, *p* < 0.01), SE (β = 0.347, *p* < 0.01) and SS (β = 0.184, *p* < 0.01).

**Conclusions:**

The HRQoL of cancer patients is correlated with HL, SS, SE and FOP. HL can directly affect HRQoL and mediate HRQoL through SS and SE. Future programs should consider HL promotion, SE improvement and SS expansion as the breakthrough point when designing targeted intervention strategies. At the same time, the importance of the impact of FOP on the HRQoL of patients with cancer should not be ignored.

## Introduction

Cancer is one of the leading causes of morbidity and mortality worldwide [1]. According to the latest global cancer burden data for 2020 released by the International Agency for Research on Cancer, there were 19.29 million new cancer cases in the world in 2020, of which 4.57 million were newly diagnosed in China, accounting for 23.7%, ranking first in the world [2, 3]. With the change in medical models and the progress of medical technology, the 5-year survival rate of malignant tumors in China has increased from 30.9% 10 years ago to 40.5% at present [4]. The survival period of cancer patients has been significantly prolonged, resulting in more attention being paid to health-related quality of life (HRQoL) during their survival years [5].

HRQoL, which is regarded as patients’ perception of the effect of illness and treatment on their current level of physical, mental, and social functioning [6, 7], has become not only a comprehensive health indicator in clinical treatments and interventions but also a way to assess the effectiveness of any disease management plan and health status [8, 9]. For example, several studies have shown that HRQoL can be used as a predictive factor of morbidity and mortality in patients with cancer [6, 10]. It has also become a core outcome measure for providing comprehensive care and supporting clinical decision-making [11]. Therefore, it is very important to study the HRQoL of cancer patients.

In an exploration of the factors affecting the HRQoL of cancer patients, in addition to the disease itself, it has been reported that HRQoL is also affected by a patient’s personal characteristics, such as health literacy (HL), self-efficacy (SE), social support (SS) and psychological characteristics [11–14].

Fear of disease progression (FOP) is one of the most common psychological symptoms in patients, which can cause cognitive and behavioral changes, increase the pain cancer patients feel, and lead to varying degrees of decreased quality of life [12, 15, 16]. When FOP becomes severe, it becomes dysfunctional [12, 16]. Certainly, the fear that cancer may develop can also be a motivating factor in promoting healthy behavior or adherence [17, 18]. Therefore, it would be important to explore the factors that moderate the impact on FOP.

SE is an important determinant of intention and behavior, which have been shown to positively influence self-management behaviors in chronic disease populations. SE can not only regulate patients’ behaviors and emotions but also encourage patients to actively monitor their own conditions and improve their quality of life. Previous investigations have shown that self-efficacy can compensate the negative impact of illness perception (IP) on FOP in cancer patients [10, 18]. Patients who had a lower self-efficacy may more likely to have a higher FOP [19]. Therefore, self-efficacy can also improve the HRQoL of patients by influencing FOP.

HL, which is a determinant of health management, is also a key driving factor promoting better HRQoL among patients with cancer [20]. HL is the degree of skills and competencies that an individual needs to obtain, process, comprehend, and use basic health information and services to make good health-related decisions, reduce their health risks, and increase their HRQoL [21]. Some studies found associations between limited HL and poor health outcomes, such as inadequate utilization of health care services, higher hospitalization rates and mortality, difficulty in making treatment decisions once diagnosed, worse skills in interpreting health information, challenges in understanding medication-related instructions, and difficulty in managing their medications [13, 20, 22, 23]. Inadequate HL has also recently been recognized as a barrier to adaptive self-management behaviors in those with long-term chronic conditions [20, 23]. There is also a significant correlation between HL and SE, which affects the HRQoL of patients [11].

SS is one factor that underlies the relationship between HL and HRQoL. People with limited HL often feel self-abasement and shame, which prevents them from seeking help. Health education can help individuals establish a good social support system by improving their HL. Some studies have shown that HL is positively correlated with SS [14, 24]. In addition, SS mediates the association of HL with HRQoL.

**Fig. 1:**
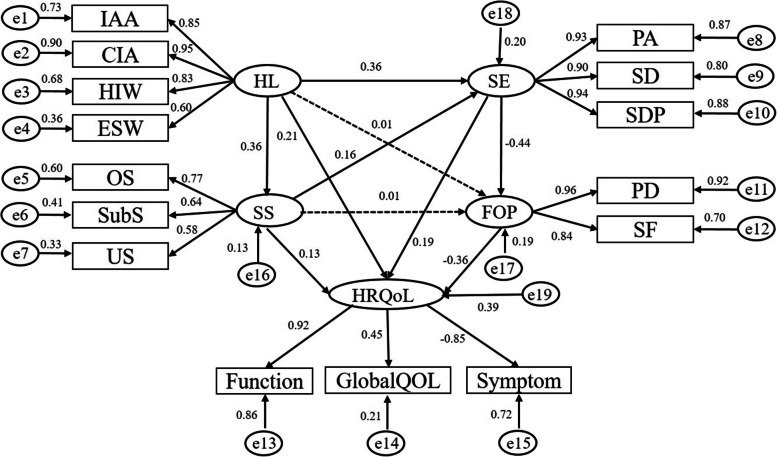
Standardized estimates of relationships and effect sizes in the structural model

Based on the above mentioned literature, previous studies have explored the relationship between any one or two of the above factors and the HRQoL of Chinese cancer patients. However, there is relatively little understanding of the synergistic effect (Synergistic effect refers to the fact that multiple factors or individuals promote, cooperate, and collaborate with each other in the process of cooperation, resulting in results that are superior to those of a single factor or individual.) or potential underlying mechanisms of these factors on HRQoL. Therefore, the purpose of this study was to a construct a structural equation model (SEM) to explore between HRQoL, HL, SS, SE and FOP and revealed the relationship between HL and HRQoL and whether this was mediated by SS, SE and FOP, which can provide theoretical basis and intervention ideas for us to better to improve the HRQoL of Chinese cancer patients. The hypothesized model is shown in Fig. 1.

## Methods

### Study design and participants

A cross-sectional survey design was used to select participants from two hospitals, one in Chengdu and another in Chongqing. The following inclusion criteria were applied: (1) at least 18 years old, (2) with a pathological diagnosis of cancer, (3) willing to provide written informed consent, and (4) without cognitive impairment or mental disorder. The study scope and purpose were explained to the patients, and written informed consent was obtained from patients who met the inclusion criteria prior to the investigation. The investigators were the nursing staff of the Oncology Department. After training, they conducted a field investigation. Answers to the questionnaire were collected either through face-to-face interviews or through self-administered questionnaires completed by the literate participants. A total of 1,800 cancer survivors were surveyed, and 1,753 responded. 4 of them were excluded because they failed to complete the questionnaires, we obtained a total of 1749 valid questionnaires.

### Instruments

#### General information questionnaire

The questionnaire included the general information of the patients, as well as blood type, occupation, monthly income, medical burden, place of residence, religious beliefs, main caregivers, family companionship, mood state, efforts made to treat their serious illness, and the decision-maker for their existing treatment plans.

#### Health literacy

HL was measured using the Health Literacy Management Scale (HeLMS) developed by Jordan et al. [25] and translated into Chinese by Sun et al. [26]. It consists of 24 questions rated on a five-point Likert scale (from 1 to 5). It was divided into four dimensions: (1) information acquisition ability (IAA, 10 items), (2) communication interaction ability (CIA, 8 items), (3) health improvement willingness (HIW, 4 items), and (4) economic support willingness (ESW, 2 items). The higher the individual and total scores, the better the health literacy.

#### Health-related quality of life

HRQoL was measured by the simplified Chinese version of the EORTC QLQ-C30 [1], which comprises 3 scales, namely, global QOL, functioning, and symptom scales. It consists of 30 questions, 28 items answered on a 4-point scale, and the two related to global QOL rated on a 7-point scale. The score of the scale is calculated by averaging the items within the scales and then linear conversion of the average score by the range method. All of the scales range from 0 to 100, with a higher score on the functional scale or overall health and a lower score on the symptom scale representing a higher quality of life. The Cronbach’s alpha for this scale was 0.72 ~ 0.89.

#### Fear of progression

FOP was measured with the simplified Chinese version of the FoP-Q-SF [27], which was developed by Wu Qiyun in Chinese from the FoP-Q-SF [28]. It contains 12 questionnaire items, and the Likert 5-level scoring method is used for each item. It includes two dimensions: physical health (PD, fear of lack of health due to illness) and social family (SF, fear of lack of family functioning due to illness). The Cronbach’s α coefficient is 0.883 for the FoP-Q-SF, and the test–retest reliability was 0.85.

#### Social support rating scale

The SSRS was developed and validated by Xiao SY in 1994 [29, 30]. It has been used widely in China. The SSRS is evaluated in three dimensions with 10 items: objective support (OS), subjective support (SubS), and utilization of support (US). Higher scores reflect better social support.

#### Self-efficacy

SE was evaluated based on the Strategies Used by People to Promote Health (SUPPH) developed by Lev and Owen [31], which was translated into Chinese by Qian Yun-hui et al. [32]. The Chinese version of the scale combines the “Alleviating stress” and “Practicing stress reduction” in the English version into one item, forming a 28 item scale divided into three dimensions: (1) positive attitude (PA, 15 items), (2) self-decision (SD, 3 items), and (3) self-decompression (SDP, 10 items) [33]. The Likert 5-level scoring method is used for each item, with scores generated from the sum of all items on the dimensions. The higher the score, the stronger the SE.

### Statistical analyses

All of the data were entered into a database in Epidata version 3.02, and all of the questionnaires were coded and double-entered by two independent professional data-entry staff. The descriptive statistics were analyzed with SPSS 22.0 software. Continuous variables are presented as the mean ± SD, and categorical data are shown as frequencies and percentages. Pearson correlation coefficients were computed to evaluate the associations between variables. A *P* value of less than 0.05 was considered statistically significant. IBM SPSS AMOS 22.0 was used to establish the SEM. HL, SE, SS, FOP and HRQoL were set as latent variables, and the corresponding entries were set as observed variables. The model was constantly refined and re-estimated to verify the model fit and to select the best-suited model. SEM was used to identify the direct, indirect, and total effects among the variables. The overall model fitness was confirmed by using fitness indices to check whether the hypothesized model fit the data well. These goodness-of-fit indices include the maximum likelihood chi-square (${X}^{2}$), comparative fit index (CFI), goodness-of-fit index (GFI), normed fit index (NFI), and root mean square error of approximation (RMSEA).

### Quality control

The survey plan and questionnaire were demonstrated and pre-investigated to identify any problems that might occur during the test and to check the reliability and validity of the scale. It was investigated by trained investigators who collected the data and randomly checked by investigators to ensure the quality of the questionnaires.

## Results

### Baseline characteristics

A total of 1800 participants were enrolled in the survey. The questionnaires were distributed to the participants, and they all submitted their answers, thereby indicating a response rate of 100%. Among the 1800 returned questionnaires, 51 questionnaires were excluded because of invalid or incomplete responses; thus, 1749 valid questionnaires were included in the analysis, indicating an effective rate of return of 97.2%. The demographic characteristics of the final participants are shown in Table 1. The responders were aged 18–91 years, with an average age of 55.34 ± 12.04 years; most were 45–60 years old. Out of the 1749 participants, 944 (54.0%) were men. The majority of the respondents were Han (94.9%). Most of them resided in urban areas (62.4%). More than half of the participants had an annual household income of less than 50,000 CNY. The majority were married (85.5%), had a primary school education or less (54.7%), their caregivers were their spouses (42.0%), had cancer stage IV (30.2%), and did not have a family history of cancer (81.3%).

**Table 1:** Demographic and other characteristics of the sample (*n* = 1749)

Characteristics	Categories	N	%
Gender	Male	944	54.0
	Female	805	46.0
Age	18 ~	314	18.0
	45 ~	780	44.6
	60 ~	655	37.5
Ethnicity	Han	1660	94.9
	Other	89	5.1
Marital status	Unmarried/Divorce/Widowed	254	14.5
	Married	1495	85.5
Education level	Below primary school	333	19.0
	Primary school	623	35.6
	High school	391	22.4
	University or higher	402	23.0
Residence	Rural	658	37.6
	Urban	1091	62.4
Annual household income	< 20,000 CNY^a^	265	15.2
	20000 ~ 49999 CNY	664	38.0
	50000 ~ 99999 CNY	552	31.6
	≥ 100000 CNY	268	15.3
Current occupational status	Not employed	377	21.6
	Employed	823	47.1
	Retired	549	31.4
Stage of Cancer	0-I	214	12.2
	II	302	17.3
	III	402	23.0
	IV	528	30.2
	Unable to judge or know	303	17.3
Family history of cancer	Yes	328	18.8
	No	1421	81.3

^a^*CNY* Chinese yuan (￥)

### Descriptive statistics for measured variables

Table 2 shows the descriptive statistics and multivariate normality of the measurement variables. The multivariate normality was verified through standard deviations, skewness, and kurtosis. We confirmed that the conditions of the normal distribution were satisfied [34]. Therefore, each factor was normally distributed, as shown in Table 2.

**Table 2:** Descriptive statistics of the measured variables (*n* = 1749)

Variables^a^	Mean	Standard deviation	Skewness	Kurtosis
IAA	40.6	8.4	-1.06	0.86
CIA	31.8	6.4	-0.95	0.98
HIW	15.8	3.5	-0.74	0.27
ESW	7.2	2.2	-0.39	-0.74
functioning	73.4	16.0	-0.52	0.72
symptom	27.5	15.4	0.36	0.63
globalQOL	53.2	19.9	-0.40	0.28
PA	45.9	11.8	0.30	-0.04
SD	9.2	2.6	0.25	-0.34
SDP	30.7	8.1	0.32	-0.18
OS	23.3	5.1	-0.41	-0.08
SubS	8.7	2.8	0.49	0.86
US	7.6	2.3	0.19	-0.49
PD	15.8	4.6	0.22	0.08
SF	15.0	5.1	0.41	-0.31

^a^*IAA* Information acquisition ability, *CIA* Communication interaction ability, *HIW* Health improvement willingness, *ESW* Economic support willingness, *QOL* Quality of life, *PA* Positive attitude, *SD* Self-decision, *SDP* Self-decompression, *OS* Objective support, *SubS* Subjective support, *US* Utilization of support, *PD* Physical health, *SF* Social family

### The correlations between HL, SE, SS, FOP and HRQoL

Table 3 documents the results of the correlation analyses of HL, SE, SS, FOP and HRQoL. The Pearson correlation analyses showed that all variables were significantly correlated with one another except for symptoms, PD and SF. However, symptoms, PD and SF were significantly correlated with each other.

**Table 3:** Correlations (r) between health literacy, self-efficacy, social support, fear of disease progression and health-related quality of life

	IAA	CIA	HIW	ESW	functioning	symptom	globalQOL	PA	SD	SDP	OS	SubS	US	PD	SF
IAA	1.000														
CIA	0.772^**^	1.000													
HIW	0.618^**^	0.749^**^	1.000												
ESW	0.481^**^	0.516^**^	0.551^**^	1.000											
functioning	0.351^**^	0.346^**^	0.348^**^	0.443^**^	1.000										
symptom	-0.287	-0.309	-0.323	-0.450	-0.767	1.000									
globalQOL	0.239^**^	0.201^**^	0.201^**^	0.297^**^	0.396^**^	-0.394	1.000								
PA	0.335^**^	0.314^**^	0.276^**^	0.321^**^	0.387^**^	-0.326	0.461^**^	1.000							
SD	0.307^**^	0.291^**^	0.250^**^	0.328^**^	0.355^**^	-0.306	0.398^**^	0.815^**^	1.000						
SDP	0.322^**^	0.321^**^	0.284^**^	0.352^**^	0.387^**^	-0.322	0.430^**^	0.852^**^	0.823^**^	1.000					
OS	0.187^**^	0.231^**^	0.246^**^	0.238^**^	0.235^**^	-0.202	0.101^**^	0.157^**^	0.171^**^	0.188^**^	1.000				
SubS	0.224^**^	0.220^**^	0.212^**^	0.217^**^	0.135^**^	-0.132	0.137^**^	0.136^**^	0.113^**^	0.133^**^	0.516^**^	1.000			
US	0.188	0.247^**^	0.228^**^	0.242^**^	0.229^**^	-0.220	0.103^**^	0.185^**^	0.204^**^	0.219^**^	0.445^**^	0.337^**^	1.000		
PD	-0.021	-0.184	-0.104	-0.228	-0.455	0.371^**^	-0.247	-0.387	-0.338	-0.364	-0.100	-0.083	-0.152	1.000	
SF	-0.199	-0.147	-0.093	-0.230	-0.412	0.342^**^	-0.226	-0.325	-0.290	-0.331	-0.035	-0.067	-0.074	0.782^**^	1.000

^**^P < 0.01

#### Measurement model

HL, SE, SS, and FOP are all associated with HRQoL, and we proposed the initial SEM. According to the research hypothesis, the path analysis diagram of the whole model was established. However, the relationship between HL, SS and FOP was not statistically significant. We therefore eliminated the direct route to fit the structural model better, which is presented in Fig. 1. The maximum likelihood ratio was used as the method of estimation, and the model fit index was used to check the degree of fit of the theoretical model to the data. The results for the model fitness are shown in Table 4. The absolute fitness indices (GFI, AGFI, and RMSEA) and value-added fitness indices (IFI, TFI, CFI) of the model met the requirements of the criteria and showed a good fit.

**Table 4:** Model fit index

Variable^a^	PGFI	PNFI	RMSEA	GFI	AGFI	NFI	RFI	IFI	TLI	CFI
Reference value	> 0.5	> 0.5	< 0.08	> 0.9	> 0.9	> 0.9	> 0.9	> 0.9	> 0.9	> 0.9
Fit index	0.644	0.742	0.072	0.943	0.917	0.950	0.936	0.955	0.942	0.955

^a^*PGFI* Parsimonious goodness-of-fit index, *PNFI* Parsimonious normed fit index, *RMSEA* Root mean square error of approximation, *GFI* Goodness-of-fit index, *AGFI* Adjusted goodness-of-fit index, *NFI* Normed fit index, *RFI* Relative fitting index, *IFI* Incremental fitting index, *TLI* Tucker-Lewis index, *CFI* Comparative fit index

The results showed that there were significant correlations between the observed variables and their corresponding latent variables (most of the regression weights were higher than 0.5). Regarding the latent variables, although the regression weights were low, HL had a significant impact on HRQoL (*r* = 0.207, *p* < 0.01) and was related to SE (*r* = 0.359, *p* < 0.01). The direct effects of SS on HL (*r* = 0.360, *P* < 0.001), SE (*r* = 0.159, *P* < 0.001) and HRQoL (*r* = 0.128, *P* < 0.001) were statistically significant. SE had a significant effect on HRQoL (*r* = 0.187, *p* < 0.01) and a negative effect on FOP (*r* = -0.440, *p* < 0.01). FOP also had a negative impact on HRQoL (*r* = -0.364, *p* < 0.01). In the SEM, the path between HL and FOP was not significant (β =  − 0.01, *p* = 0.699), and the path between SS and FOP was also not significant (β =  − 0.01, *p* = 0.659).

Table 5 presents the indirect, direct, and total effects of the various latent variables. According to the analysis conducted with the latent variables, the effect of HL indirectly affected HRQoL through SE (coefficient = 0.124, *p* < 0.01). The indirect effect of SS on HRQoL was 0.175 through the chain mediating effect of HL and SE. Similarly, for the effect of SE that indirectly affected HRQoL, the coefficient was 0.160. We found that the indirect effect of SE on HRQoL was stronger than the direct effect. For cancer patients, FOP is the most influential factor of their quality of life, followed by SE. However, there is little difference in the degree of influence of these factors.

**Table 5:** Factor effect breakdown of health-related quality of life

Relationship between variables^a^	Direct Effects	Indirect Effects^b^	Total Effects
H1: HL → SS	0.360	0.000	0.360
H2: HL → SE	0.359	0.057	0.416
H3: HL → HRQoL	0.208	0.191	0.398
H4: SS → SE	0.159	0.000	0.159
H5: SS → HRQoL	0.128	0.055	0.184
H6: SE → FOP	-0.440	0.000	-0.440
H7: SE → HRQoL	0.187	0.166	0.347
H8: FOP → HRQoL	-0.364	0.000	-0.364

^a^*HL* Health literacy, *SS* Social support, *SE* Self-efficacy, *HRQoL* Health-related quality of life, *FOP* Fear of disease progression

^b^*HL-SS-SE* HL-SS-HRQoL, HL-SE- HRQoL, HL-SE-FOP- HRQoL, *SS-SE-FOP-HRQoL* SE-FOP-HRQoL

## Discussion

This study aimed to better understand the complex factors that influence the HRQoL of cancer patients. First, we developed a comprehensive model that illustrates the relationships between the multiple variables and HRQoL in cancer patients. Second, we examined the potential mechanisms and interactions among these factors by SEM. We found that HL, SE and SS could positively affect the HRQoL of cancer patients, whereas FOP had a negative impact on the HRQoL of cancer patients. Therefore, this study opens a new door for improving the HRQoL of cancer patients, emphasizing the role of SS, SE, HL and other factors. We also illustrated how closely they relate to HRQoL.

Compared with SS, SE and FOP, HL promoted HRQoL in cancer patients and had the largest effect coefficient. Extensive studies have also shown that there is a significant positive correlation between HL and HRQoL [6, 8, 11]. They also found that, similar to this study, health literacy was an important predictor of QOL [20]. People with high HL were more likely to acquire relevant health knowledge and develop good health behavior [29]. The author further found that the demanding nature of the cancer treatment process, which required individuals to understand the potential benefits and potential side effects of their treatment, were highly correlated to their HL [35].

Our model suggests that HL not only directly enhances HRQoL but also exerts indirect effects by improving patients’ SS and SE. The stronger the SS, the higher the patient’s HRQoL, which has been found in similar studies [14, 36]. SS networks can guide patients to make more use of various social resources, cooperate with various SSs from family, friends and society, and enhance the ability of individuals to manage their own health by enhancing their confidence and motivation to improve their HRQoL [37]. In other words, SS has a positive effect on the HRQoL pathway, which was also confirmed in our study.

In addition, SS can reduce the negative impact of low HL [14]. Individuals need help from family and friends when processing health-related information. This was confirmed in our study. The indirect effect of HL on HRQoL through SS and SE was 0.191. Relevant studies have further confirmed that improving HL can enable patients to use SS to improve their care ability [38].

SE will affect people’s behavior and health trends. One study demonstrated that there was a positive effect of SE on HRQoL [39]. Patients with an increased sense of SE may feel more capable of dealing with their situation. One study emphasized that direct guidance given for improving the SE of individuals has a positive effect on managing their disease and treatment [11]. Knowledge is a factor contributing to the enhancement of SE. Studies have shown that higher levels of HL contribute to higher attainment of knowledge and a higher SE [40].

Our results suggested that SE can reduce FOP and indirectly affect HRQoL. Thus, SE can directly and indirectly affect HRQOL. Researchers have found that expectations about a person’s ability to handle difficult and challenging situations affects their emotional responses [41]. These results may be helpful in considering techniques to enhance SE as part of the treatment of disease-related anxiety. At the same time, as SE increases, the relationship between more severe physical symptoms and lower functioning and emotional health weakens [18].

In our study, there was no significant relationship between FOP and HL or SS. This is inconsistent with the results of other related studies [42, 43]. The study by Marius Haack et al. found that better HL was associated with decreased FOP [42]. At the same time, they also noted that HL knowledge of cancer and certain aspects of one’s own physical condition were associated with increased anxiety. Regarding SS and FOP, the relevant literature reported that SS could reduce FOP [44–46], which was also found in our study, but it was not statistically significant. It may be that FOP is influenced by many factors [47]. Therefore, we will continue to explore the relationships between SS, HL and FOP in future studies.

### Strength and limitations

There are several limitations in this paper. First, the participants were potentially eligible patients recruited from two hospitals using convenience sampling methods, so the population was not well represented. Additional investigations with a random sampling method should be designed or participants should be recruited from the community. Second, the data analyzed were cross-sectional and self-reported. Therefore, no conclusions can be drawn about causation. In the future, longitudinal studies can be designed to explore the causal relationships and synergistic effects among these variables. Third, the relationship among the variables may be inflated due to response bias. Because certain variables react more strongly in special environments, such as hospital. Certainly, many variables are being studied, including HL, SE, SS and FOP associated with HRQoL of cancer. This is the strength of this research. What’s more, we have established the relationship between these variables for the first time, which can provide theoretical basis and intervention ideas for us to better improve the quality of life of cancer patients.

## Conclusions

The HRQOL of cancer patients is poor and affected by many factors. This study provides a unique perspective to explore the relationships between the HRQoL of cancer patients and HL, SE, SS and FOP. The SEM of HRQoL works well. HL, SE, SS, and FOP all have an impact on HRQoL, with HL having the greatest impact. For HL to be improved, patients should be prioritized for evaluation of HL prior to intervention to reasonably match their coping needs, study the factors that affect HL, and improve the level of health literacy. At the same time, we should also pay attention to the moderating effects of SS and SE. Medical staff should consider how to improve HL, SE and targeted SS, matched to their individual needs, and they should strive to reduce the patients’ FOP, ultimately improving their quality of life. Therefore, during the intervention, a multimodal intervention plan needs to be developed to improve other outcomes that may have an impact on causal pathways. For example, by improving health literacy, personal self-efficacy can be improved, thereby improving the quality of life.

## Data Availability

The datasets used and/or analyzed during the current study are available from the corresponding author on reasonable request.

## Abbreviations

HL: Health literacy
SE: Self-efficacy
SS: Social support
FOP: Fear of disease progression
HRQoL: Health-related quality of life
SEM: Structural equation model
IAA: Information acquisition ability
CIA: Communication interaction ability
HIW: Health improvement willingness
ESW: Economic support willingness
PD: Physical health
SF: Social family
OS: Objective support
SubS: Subjective support
US: Utilization of support
SUPPH: Strategies used by people to promote health
PA: Positive attitude
SD: Self-decision
SDP: Self-decompression
CFI: Comparative fit index
GFI: Goodness-of-fit index
NFI: Normed fit index
RMSEA: Root mean square error of approximation
PGFI: Parsimonious goodness-of-fit index
PNFI: Parsimonious normed fit index
AGFI: Adjusted goodness-of-fit index
RFI: Relative fitting index
IFI: Incremental fitting index
TLI: Tucker-Lewis index

## References

[1] Xia J (2019). Relationship between health literacy and quality of life among cancer survivors in China: a cross-sectional study. BMJ Open.

[2] Sung H (2021). Global Cancer Statistics 2020: GLOBOCAN Estimates of Incidence and Mortality Worldwide for 36 Cancers in 185 Countries. CA Cancer J Clin.

[3] Wild CP, W.p.E, Stewart BW. World Cancer Report: Cancer Research for Cancer Prevention, World Health Organization (WHO), 2020. https://www.iarc.fr/cards_page/world-cancer-report.

[4] The five-year survival rate of malignant tumor in China has increased to 40.5 (Chinese). 2021. http://news.cctv.com/2021/04/27/ARTIHGB2q5QEjNxLROHfGP1h210427.shtml?ivk_sa=1023197a.

[5] Ghislain I (2016). Health-related quality of life in locally advanced and metastatic breast cancer: methodological and clinical issues in randomised controlled trials. Lancet Oncol.

[6] Magon A (2021). Trajectories of health-related quality of life, health literacy, and self-efficacy in curatively-treated patients with esophageal cancer: a longitudinal single-center study in Italy. J Patient Exp.

[7] Cella DF, Tulsky DS (1993). Quality of life in cancer: definition, purpose, and method of measurement. Cancer Invest.

[8] Zhang Q (2021). The effect of high blood pressure-health literacy, self-management behavior, self-efficacy and social support on the health-related quality of life of Kazakh hypertension patients in a low-income rural area of China: a structural equation model. BMC Public Health.

[9] Jayasinghe UW (2016). The impact of health literacy and life style risk factors on health-related quality of life of Australian patients. Health Qual Life Outcomes.

[10] Avery KNL (2018). Development of a core outcome set for clinical effectiveness trials in esophageal cancer resection surgery. Ann Surg.

[11] Ozkaraman A (2019). The effect of health literacy on self-efficacy and quality of life among Turkish cancer patients. J Pak Med Assoc.

[12] Goebel S, Mehdorn HM (2019). Fear of disease progression in adult ambulatory patients with brain cancer: prevalence and clinical correlates. Support Care Cancer.

[13] Humphrys E (2019). The influence of health literacy on the timely diagnosis of symptomatic cancer: a systematic review. Eur J Cancer Care (Engl).

[14] Kobayashi R, Ishizaki M (2020). Relationship between health literacy and social support and the quality of life in patients with cancer: questionnaire study. J Particip Med.

[15] Herschbach P, Dinkel A. Fear of progression. Recent Results Cancer Res. 2014;197:11–29. 10.1007/978-3-642-40187-9_2 24305766

[16] Simard S (2013). Fear of cancer recurrence in adult cancer survivors: a systematic review of quantitative studies. J Cancer Surviv.

[17] Simon R (2014). Adherence to adjuvant endocrine therapy in estrogen receptor-positive breast cancer patients with regular follow-up. Can J Surg.

[18] Shim EJ, Lee JW, Min YH (2018). Does depression decrease the moderating effect of self-efficacy in the relationship between illness perception and fear of progression in breast cancer?. Psychooncology.

[19] Bugaj TJ (2023). Couples coping with advanced prostate cancer: an explorative study on decision-making preferences, self-efficacy and fear of progression. World J Urol.

[20] Wei CW, Wu ML, Tung HH (2021). Relationships between health literacy and quality of life among survivors with breast cancer. Int J Nurs Pract.

[21] Berkman ND, Davis TC, McCormack L (2010). Health literacy: what is it?. J Health Commun.

[22] Clarke N (2021). Health literacy impacts self-management, quality of life and fear of recurrence in head and neck cancer survivors. J Cancer Surviv.

[23] Nilsen ML (2020). Health literacy: Impact on quality of life in head and neck cancer survivors. Laryngoscope.

[24] Lee SYD (2009). Health literacy, social support, and health status among older adults. Educ Gerontol.

[25] Jordan JE, Buchbinder R, Osborne RH (2010). Conceptualising health literacy from the patient perspective. Patient Educ Couns.

[26] Sun HL, Peng H, Fu H (2012). The reliabililty and consistency of health literacy scale for chronic patients. Fudan Univ J Med Sci.

[27] Wu Q, Ye Z, Li LI, Liu P (2015). Reliability and validity of Chinese version of fear of progression questionnaire-short form for cancer patients. Chinese J Nurs.

[28] Mehnert A (2006). Fear of progression in breast cancer patients–validation of the short form of the Fear of Progression Questionnaire (FoP-Q-SF). Z Psychosom Med Psychother.

[29] Li Y (2020). Health literacy, social support, and care ability for caregivers of dementia patients: Structural equation modeling. Geriatr Nurs.

[30] Xiao S (1994). Theoretical foundation and research application about the social support rating scale. J Clin Psychiatry.

[31] Lev EL, Owen SV (1996). A measure of self-care self-efficacy. Res Nurs Health.

[32] Qian HJ, Yuan C (2011). The reliability and validity of Chinese version of strategies used by people to promote health. Chin J Nurs.

[33] Tao L (2020). Exercise adherence in breast cancer patients: a cross-sectional questionnaire survey. Medicine (Baltimore).

[34] Lim SH, Cho IY (2022). A structural equation model of developing a partnership between pediatric nurses and parents of children with cancer in South Korea. J Pediatr Nurs.

[35] Kugbey N, Meyer-Weitz A, Oppong Asante K (2019). Access to health information, health literacy and health-related quality of life among women living with breast cancer: depression and anxiety as mediators. Patient Educ Couns.

[36] Robb C (2013). Health and personal resources in older patients with cancer undergoing chemotherapy. J Geriatr Oncol.

[37] Wang Y (2022). Association among high blood pressure health literacy, social support and health-related quality of life among a community population with hypertension: a community-based cross-sectional study in China. BMJ Open.

[38] Kyriazidou E (2022). Health-related quality of life and social support of elderly lung and gastrointestinal cancer patients undergoing chemotherapy. SAGE Open Nurs.

[39] Kueh YC (2015). Modelling of diabetes knowledge, attitudes, self-management, and quality of life: a cross-sectional study with an Australian sample. Health Qual Life Outcomes.

[40] Kim K (2018). Decisional balance and self-efficacy mediate the association among provider advice, health literacy and cervical cancer screening. Eur J Oncol Nurs.

[41] Melchior H (2013). Self-efficacy and fear of cancer progression during the year following diagnosis of breast cancer. Psychooncology.

[42] Haack M (2020). Quality of life and fear of disease progression are associated with aspects of health literacy in men with prostate cancer from Germany. Support Care Cancer.

[43] Lardas M (2017). Quality of life outcomes after primary treatment for clinically localised prostate cancer: a systematic review. Eur Urol.

[44] Ban Y (2021). The effect of fear of progression on quality of life among breast cancer patients: the mediating role of social support. Health Qual Life Outcomes.

[45] Zhong M (2022). The mediating effects of resilience on perceived social support and fear of cancer recurrence in glioma patients. Psychol Res Behav Manag.

[46] Zheng W, Hu M, Liu Y (2022). Social support can alleviate the fear of cancer recurrence in postoperative patients with lung carcinoma. Am J Transl Res.

[47] Niu L, Liang Y, Niu M (2019). Factors influencing fear of cancer recurrence in patients with breast cancer: evidence from a survey in Yancheng, China. J Obstet Gynaecol Res.

